# The Effect of Relational Embeddedness, Absorptive Capacity, and Learning Orientation on SMEs’ Competitive Advantage

**DOI:** 10.3389/fpsyg.2020.01505

**Published:** 2020-07-24

**Authors:** Guo-Song Wu, Michael Yao-Ping Peng, Zhong Chen, Zongmin Du, Muhammad Khalid Anser, Wen-Xuan Zhao

**Affiliations:** ^1^Huzhou University, Huzhou, China; ^2^School of Economics & Management, Foshan University, Foshan, China; ^3^School of Digital Economics, Guilin University of Electronic Technology, Guilin, China; ^4^School of Economics, Fujian Normal University, Fuzhou, China; ^5^Business School, University of National and World Economy, Sofia, Bulgaria; ^6^School of Public Administration, Xi’an University of Architecture and Technology, Xi’an, China; ^7^Graduate Institute of Management, Chang Gung University, Taoyuan, Taiwan

**Keywords:** absorptive capacity, competitive advantage, learning orientation, relational embeddedness, social capital

## Abstract

The combination of external new knowledge and organizational capability has become a cornerstone in the internationalized enterprises. Sources of knowledge acquisition are among the most valuable resources that an internationalized firm can achieve competitive advantage. This study aims to explore this phenomenon between knowledge sources and competitive advantage in the context of internationalized small and medium enterprises (SMEs) mainly by a quantitative approach. Based on contextual nature of resource–capability–competitive, absorptive capacity has long been a critical construct in organizational studies, as well as referring relational embeddedness as potential drivers of absorptive capacity, which is moderated by learning orientation. This study also investigates the mediating effect of absorptive capacity in terms of knowledge absorption and integration on international competitive advantage. To gain insights and provide essential implications for internationalized SMEs from the global economy, this study adopted purposive sampling to collect 211 valid responses. Via the partial least-squares structural modeling, the relationships among relational embeddedness, potential and realized absorptive capacity, and competitive advantage were measured. The results suggest that relational embeddedness positively influences potential and realized absorptive capacity with moderating effect of learning orientation. Potential and realized absorptive capacities have full mediating effect between relational embeddedness and competitive advantage. This study concludes insights and implications for research and practice.

## Introduction

Much attention has been given to competitive advantage by scholars, especially in strategies and organizational management. There are also many insights on competitive advantage (McGrath, 2013; Rui et al., 2016; Delery and Roumpi, 2017; Harrigan and Diguardo, 2017). As shown in previous studies, the competitive advantage shows that firms respond to competitors’ competitive behavior effectively and superiorly by adapting and adjusting to their own resources in facing dynamic environment changes (Li and Zhou, 2010; Delery and Roumpi, 2017). However, because of the continuous changes in the foreign market and industry competition environment, internationalized firms cannot persistently respond to competitive behaviors within the industry, especially for small and medium enterprises (SMEs). Some scholars believe that core competitiveness and capabilities should be maintained in order to achieve sustainable competitive advantages (Saranga et al., 2018). The aforementioned is the local perspective of competitive advantage, but it is more difficult to be maintained and established from the international perspective (Li and Zhou, 2010). Internationalized SMEs not only need to overcome the competition from the local industry, but also face the risks brought by the global companies’ competitive behaviors and the heterogeneity of diversified overseas markets, which highlights the importance of international competitive advantages (Kwak et al., 2018). Therefore, this study combines internationalization and organizational learning theories to examine the key antecedents of internationalized SMEs’ competitive advantage, merely studying several main effects (Saranga et al., 2018) and some specific variables of competitive advantages (Li and Zhou, 2010) for the internationalized extension of SMEs (Kwak et al., 2018).

This study summarizes the existed literature on competitive advantage by analyzing the effect of two factors that can enhance competitive advantage: relational embeddedness (RE) and knowledge absorptive capacity (AC). However, there is still an interesting research issue in the social capital literature (Aribi and Dupouët, 2015; García-Villaverde et al., 2018) about the reason for verifying the impact of knowledge acquisition and knowledge learning on the effect of the relational dimension of social capital. Social capital view has increasingly been applied in the interdisciplinary field, especially in management and education (García-Villaverde et al., 2018). In the distinction of social capital dimensions, Nahapiet and Ghoshal (1998) have divided them into relational, cognitive, and structural dimensions. Each dimension has a different focus (Mitrega and Pfajfar, 2015; Knight and Liesch, 2016; Pinho and Prange, 2016; Liu et al., 2018). However, relational dimension will be conducive to discussing the relevance in this study to understand how internationalized SMEs gain valuable knowledge source to achieve competitive advantages. According to previous studies of scholars, this study adopts RE as a significant variable for measuring relational dimension (Dhanaraj et al., 2004). Specifically, Riikkinen et al. (2017) indicated that it is not known whether international companies differ from domestic company in terms of knowledge process of resource–capability–competitive. Their results indicated that there is an existing significant difference between international and domestic domains and found that AC plays a key role in their implementation of knowledge learning process. For this reason, this study aims to investigate how internationalized SMEs foster their own AC and then enhance competitive advantage.

In order to seek competence and business boundaries, most internationalized SMEs generally acquire new knowledge introduced from network partners and then integrate the existed knowledge with the new knowledge in order to develop specific own knowledge that belongs to the internationalized SMEs (Liao et al., 2016). From this perspective, McEvily and Marcus (2005) argued that previous studies have typically neglected the process perspective. In this way, through the knowledge acquisition and the renewing of existed knowledge, SMEs can facilitate better learning and creation and thus maintain a sustainable competitive advantage (Liao and Hu, 2007; Huang and Yu, 2011). These processes are referred to as perspectives of organizational learning and AC (Sun and Anderson, 2012; Larrañeta et al., 2016). The development of AC reinforces, complements, and emphasizes existed knowledge base possessed by the internationalized SME (Lane et al., 2006), promoting the maintenance of competitive advantage. Thus, the promotion of well-established capacities will facilitate SMEs to take greater advantage of recognizing, assimilating, and exploiting the knowledge from their network connectedness and then to drive innovativeness and proactiveness of internationalized SMEs (Atuahene-Gima and Murray, 2007).

In the previous literatures, scholars have different views on the operationalization of AC. Specifically, some scholars believe that AC is a phase of knowledge processing, which can be divided into knowledge acquisition, assimilation, transformation, and exploitation (García-Villaverde et al., 2018), whereas some other scholars divide AC as potential (PAC) and realized AC (RAC) (Kang and Lee, 2017; Flor et al., 2018; Limaj and Bernroider, 2019). But there is a similarity in these views. All of them hold that adsorptive capacity is a high-order dimension that is composed of diverse measuring variables (Kang and Lee, 2017; Flor et al., 2018; García-Villaverde et al., 2018; Limaj and Bernroider, 2019). Literature suggests that AC represents closely related to specific capabilities firms can utilize to maintain their competitive advantage (Lane et al., 2006). This study follows Kang and Lee’s (2017), Flor et al. (2018) conceptualization of AC, which divide into PAC and RAC. With regard to this, this study aims to explore the role of AC in the research framework and provides three reasons. First, while lots of previous studies considering these dimensions have advanced our understanding of the original concept, there are few empirical studies specifically exploring figuration and development of AC (Kang and Lee, 2017; Limaj and Bernroider, 2019). In addition, there are still few studies to explore the role of AC in the context of internationalized SMEs (Limaj and Bernroider, 2019), which is significant discussing how important it is for internationalized SMEs to adapt to rapidly changing environment while operating with limited resources. Third, there are few empirical studies to explore the concept of ambidexterity (combination of RAC and PAC), which is particularly important in the context of internationalized SMEs (Heavey et al., 2015; Broersma et al., 2016; Rafailidis et al., 2017).

Likewise, some previous studies suggest that specific culture can decrease or increase the development of capacity in organizations (Naranjo−Valencia et al., 2011; García-Villaverde et al., 2018), given the specific cultures in any organization reflected by different sets of values beliefs, assumptions, and values (Cameron and Quinn, 2006; Yilmaz and Ergun, 2008). The development of AC in organizations can be enhanced only when appropriate culture is cultivated under suitable conditions (García-Villaverde et al., 2018). According to the organizational learning theory, organizations must be equipped with similar knowledge base and positive attitudes toward knowledge learning in the process of acquiring external knowledge and information, so as to transfer knowledge into elements of cultivating AC. In particular, scholars argue that learning orientation (LO) is the most significant internal learning culture of organizations in the process that firms convert and internalize external knowledge and information. Prior studies, however, seldom discuss the function of LO and its role in knowledge conversion under the framework of AC development. Thus, this study aims to refer LO culture as a moderator and discuss the effect on relationship among RE, AC, and competitive advantage.

According to the above arguments, this study aims to propose three contributions based on the following theoretical insights: (1) applying organizational learning theory to explore how internationalized SMEs enhance AC through knowledge acquisition with their foreign partners in the international context; (2) dividing AC into PAC and RAC from the perspective of high-order dimension and verifying the relevance among RE, PAC, RAC, and competitive advantage; (3) adding LO from organizational culture perspective to research framework to explore whether internationalized SMEs with higher LO strengthen their PAC and RAC.

## Literature Review and Hypotheses Development

### Organizational Learning and Competition Advantage

Organizational learning theory has attracted much attention from a majority of scholars in studies of management theory, and the theory has been enriched combined with knowledge domain in different fields (Liao et al., 2016). Scholars point out that knowledge is an important factor for firm capacity and boundary. To prevent imitation and replication of competitors, most firms engage in acquiring new knowledge from outside, or learning new knowledge from partner enterprises, and integrating new knowledge and existing knowledge, thus improving the efficiency of internal process or the development of new products (Liao et al., 2016). On the basis of organization learning, more new knowledge is acquired, and existing knowledge is updated more frequently, so that firms can optimize their learning and innovative behaviors to obtain sustainable competitive advantages (Liao and Hu, 2007; Huang and Yu, 2011; Larrañeta et al., 2016).

The literatures on firm competitive advantage from the perspective of organizational learning have been growing. These literatures have been empirically supported, no matter from the perspective of the internationalized SMEs, the network type of firms, or the influence of knowledge flow on competitive advantage (Li and Zhou, 2010; Rui et al., 2016). However, the persistence of competitive advantages (Harrigan and Diguardo, 2017) and how large firms or high-tech industries gain competitive advantages in the process of industrial competition (Kwak et al., 2018) were rarely discussed. The competitive advantage of internationalized SMEs is different from that of other firm size. Most of them enhance their innovation capability and development of new products through mergers and acquisitions or international cooperation and provide new products and services that are superior to new markets and customers. The competitive advantage under internationalization also needs to be incorporated with institutionalized competitive advantages based on past differentiation and cost leadership to further understand the effectiveness and capability growth of the internationalized SMEs (Li and Zhou, 2010).

### Absorptive Capacity

There is a lack of understanding of how to assess valuable knowledge from the external environment, how to shape the knowledge creation process of firms, and how to strengthen the combination of existed knowledge base with external knowledge and enhance internal innovation and performance by conversion. These are all related to the AC (Cohen and Levinthal, 1990). According to the definition of AC proposed by Lane et al. (2006), Peng and Lin (2019) refer to AC as “the capacity of firms to effectively utilize external knowledge through three consecutive processes, including (1) to identify and understand potential valuable knowledge outside the firm through exploratory learning; (2) to absorb new valuable knowledge through transformative learning; (3) to use the absorbed knowledge to create new knowledge and commercial consequence through exploitative learning” (p. 7).

Following the claims of Escribano et al. (2009), AC relies on firms’ existed knowledge assets and emphasizes recognizing, integrating, and utilizing new knowledge. The knowledge assets are mostly embedded in procedures, personnel, and products (Peng and Lin, 2019). In terms of conceptualization, most scholars indicated that AC is a higher-order construct (Zahra and George, 2002; Flatten et al., 2011; Kang and Lee, 2017; Limaj and Bernroider, 2019). Absorptive capacity is a composite of four capabilities, including acquisition, assimilation, transformation, and application. The acquisition and assimilation constitute the PAC. The transformation and application constitute the RAC (Zahra and George, 2002; Limaj and Bernroider, 2019). Representatively, the main functions of PAC are to acquire and digest knowledge acquired from outside and further create new knowledge through internal process; the function of RAC is to convert internal knowledge and apply it to the response to environmental changes (Zahra and George, 2002; Camisón and Forés, 2010; Flatten et al., 2011; Limaj and Bernroider, 2019).

Some scholars argued that development of AC may explain innovation, performance, or competitive advantage through theoretical framework from different perspectives, including organizational culture (Limaj and Bernroider, 2019), social capital (García-Villaverde et al., 2018), and innovation (Kang and Lee, 2017; Flor et al., 2018). Specifically, organizational learning theory contributes to explain reasons, antecedents, and insights under which AC creates value and advantage (Zahra and George, 2002; Jansen et al., 2005; Lane et al., 2006; Limaj and Bernroider, 2019). Potential AC plays a leading role in the process of firms creating values. The main task of PAC is to acquire external knowledge and digest them into internally recognized knowledge. In the model of input–process–output, the quality and value of knowledge must be verified. Therefore, firms with better PAC will be able to acquire more new and implicit knowledge and information with low repeatability (Liao et al., 2016). When this knowledge is introduced during the internal knowledge integration and knowledge creation, firms will be able to create new knowledge with more values than competitors and improve internal process management and efficiency of routines, thereby enhancing their competitive advantages. Therefore, this study proposes the following hypothesis:

**Hypothesis 1 (H1):** Potential absorptive capacity positively affects competitive advantage.

However, RAC is not consistent in all units of an organization, because the building and accumulation of RAC usually need various knowledge to produce new ideas and innovative design (Escribano et al., 2009; Broersma et al., 2016; Peng and Lin, 2019). Without RAC, it fails to transfer knowledge from other units (Lane et al., 2006). Moreover, the internationalized process of firms is a type of organizational learning. The knowledge and information in the overseas market should be to integrate with the RAC to facilitate internationalized SMEs to clarify a specific strategic position and confirm needs of global customers, thereby strengthening the application (Eisenhardt and Martin, 2000; Morgan et al., 2009; Rothaermel and Alexandre, 2009; Peng and Lin, 2019). Besides, environmental changes in international market are more intense than that in local market. Internationalized firms need to be equipped with enough information and capacities to adapt to changes of competitors and markets. Internationalized SMEs with more RAC are able to adjust internal knowledge according to changes in external environment and propose specific strategic behaviors to gain dominant strategic position (Liao et al., 2016). In addition, scholars also indicate that RAC is able to promote innovative behavior of firms and accelerate the process of knowledge creation, thereby leading to competitive activities of competitors (Limaj and Bernroider, 2019). Therefore, this study proposes the following hypothesis:

**Hypothesis 2 (H2):** Realized absorptive capacity positively affects competitive advantage.

When organizations face basic problems, they often use adequate PAC to ensure adaptable viability. However, organizational resources should be allocated to RAC sufficiently to ensure future viability (Peng and Lin, 2019). Potential AC and RAC are effective drivers for growing competitive advantage. Potential AC can enhance the productivity of existed knowledge in several contexts, whereas RAC can facilitate organizational capabilities to integrate new knowledge with the original knowledge stock (Peng and Lin, 2019), thereby enabling internationalized firms to create new goods to fight against competitors’ activities (Koryak et al., 2018). Most of previous studies verify the effect of RAC and AC on dependent variables from the first-order level, such as innovation (Flor et al., 2018; Limaj and Bernroider, 2019), innovative behavior (Kang and Lee, 2017), learning sustainability (Riikkinen et al., 2017), and customer acquisition and retention. Both capacities are important variables measuring AC. Although this study figures out the relationship of hierarchical order between the two capacities, that is, PAC has a positive effect on RAC (Riikkinen et al., 2017), few studies further discuss the joint effect caused by the concurrence of activities subject to PAC and RAC or put emphasis on the interaction of activities. The subject of this study can be considered as ambidexterity in research about organizational management.

Gibson and Birkinshaw (2004) refer to Tushman and O’Reilly’s (1996) findings on the concept of ambidexterity, using the “juggler” metaphor to depict an ambidextrous organization’s goal of efficiency and innovation in primary markets on the one hand (Peng et al., 2019), whereas on the other hand, such companies can carry out rapid and flexible explorations of emerging markets by developing new goods (Andriopoulos and Lewis, 2010). The concept of ambidexterity represents the combination of both PAC and RAC via differentiated groups, units, or functions (Zhang and Chen, 2013; Peng et al., 2019; Peng and Lin, 2019). However, ambidextrous organizations implement inspection activities in a fast and flexible way to provide new goods in global markets (Andriopoulos and Lewis, 2010; Vahlne and Jonsson, 2017; Peng and Lin, 2019).

The ambidexterity formed by the PAC and RAC will help firms focus on the development of knowledge acquisition and knowledge creation simultaneously and reduce the allocation risk from overfocusing on PAC and RAC (Heavey et al., 2015; Broersma et al., 2016; Vahlne and Jonsson, 2017; Luca et al., 2018). Furthermore, several scholars claimed that the ambidexterity is referred to as a structural mode (Tushman and O’Reilly, 1996; Andriopoulos and Lewis, 2010; Peng and Lin, 2019; Peng et al., 2019), which promotes structural equation modeling (SEM) to establish various organizational structures to deal with contradictions and opposites through the differentiation (Peng and Lin, 2019). By using both PAC and RAC and thus making utilization of existed knowledge and resources, internationalized SMEs can detect relevant knowledge/resources more easily and understand the situation more thoroughly (Rafailidis et al., 2017), thus leading to the more effective reconfiguration of existing knowledge/resources while promoting PAC and RAC to leverage existing valuable resources and overcome boundaries to drive future market opportunities (Spyropoulou et al., 2018). Thus, this study proposed the following hypothesis:

**Hypothesis 3 (H3):** Ambidexterity of PAC and RAC positively correlates with competitive advantage.

### Relational Embeddedness

The closer the relationship between firms and their network members, the more advanced their network relationship is (Evangelista and Hau, 2009). However, as the relationships between firms and their network members are established through the accumulation of long−term interactions, they will develop norms and trust and share value and mutual recognition during this process (Moran, 2005). Gilsing and Duysters (2008) regarded it as the relational dimension of social capital (i.e., RE). Moran (2005) believes that RE refers to the network cohesion and stresses the direct cohesion of ties, which can help network members obtain detailed information (Gilsing and Duysters, 2008). Dhanaraj et al. (2004) claimed that organizations could exchange information and knowledge through market, hierarchy, and a hybrid form, of which international joint venturing is a typical example of hybrid form, and they expatiate the relationships between their organizations with the three factors in RE, that is, strength of ties, trust, and shared system (Nielsen, 2005; Park and Glaister, 2009).

The legal contract between the enterprises clearly shows the boundaries of their co-operations, which restrict the development of relationships between them, but the embedded social relationship can break through these boundaries (Gilsing and Duysters, 2008; Chung, 2012). Relational embeddedness can overcome these obstacles and reduce the cost of knowledge acquisition by the free exchange of knowledge created, thus facilitating learning (Luca et al., 2018). Hansen (1999) pointed out that weak ties are conducive to searching out the necessary knowledge and information quickly, but they will impede knowledge transfer when the knowledge is of high complexity and difficult to be made explicit (Evangelista and Hau, 2009). Tiwana (2008) holds that shared values among network members will enable enterprises to absorb easily the ideas and thoughts of their counterparts, contributing to the transmission and integration of tacit knowledge, reducing mistrust and uncertainties, and helping both parties together get involved in the resolution of alliance issues (McEvily and Marcus, 2005). If an enterprise and its partners have a more close relationship, both parties would be more willing to transfer their respective tacit or complex knowledge to each other (Moran, 2005; Nielsen, 2005; Park and Glaister, 2009; Squire et al., 2010).

Therefore, enterprises with good RE are able to compare their existing knowledge with new network knowledge (Gilsing and Duysters, 2008; Heavey et al., 2015); by incessant, repeated reflections, such enterprises can know about the content and quality of the knowledge, resolve problems together with their customers and suppliers related, and then find out their own tacit knowledge (Park and Glaister, 2009; Luca et al., 2018). As stated above, firms acquire, convert, and apply external knowledge through RE. This interorganizational learning process can combine external and internal knowledge and strengthen existing PAC and RAC (García-Villaverde et al., 2018). Therefore, hypotheses are made in this study as follows:

**Hypothesis 4 (H4):** Relational embeddedness positively affects PAC.
**Hypothesis 5 (H5):** Relational embeddedness positively affects RAC.

### The Mediating Role of AC

Many prior studies point out that firms with intense network relationship will be able to acquire rarer and more valuable knowledge such as patents and intellectual properties. This knowledge will be conducive to firms achieving better performance in innovation (Teece, 2007; Kotabe et al., 2017), development of new products (Audretsch et al., 2015, 2016), and market development, thereby promoting the gain of competitive advantages. However, Scuotto et al. (2017) indicate that, for the success of an innovative SME, it is not sufficient to acquire and combine knowledge that is capable of bringing together new ideas, and a social network is also required in order to support the innovation process in the long term. In other words, firms must internalize acquired information and knowledge through RE, integrate external knowledge and internal AC, and convert them into proprietary abilities and knowledge of firms and further apply them to competitive behaviors to gain competitive advantages. This knowledge acquisition and application process facilitates a combination of fragmented knowledge within a firm and leads to competitive behavior (Scuotto et al., 2017). Hence, AC in conjunction with RE results in the following hypothesis:

**Hypothesis 6 (H6):** The relationship between RE and competitive advantage is mediated by AC (PAC and RAC).

### Learning Orientation

According to Baker and Sinkula (1999), LO is an advanced learning concept and a sequence of organizational values that represent the tendency of a firm of creating and using knowledge. Organizations are motivated by these values to improve their paradigms and speed up paradigm transfer (Miner et al., 2001). In consequence, the organizational effectiveness is upgraded, and the heterogeneity and range of SMEs’ knowledge are increased. Sinkula et al. (1997) explained LO from three dimensions: (1) commitment to learning, which signifies the promise of an organization to promote a learning culture and place value on the learning activity; (2) open-mindedness, which is linked to the notion of unlearning (i.e., questioning, assumptions, beliefs, and routines); and (3) shared vision, focusing on the learning that cultivates energy, makes learning commitment, and achieves purpose among members. Learning orientation defines the capabilities of an organization for creating, spreading, and utilizing knowledge (Sinkula et al., 1997). Moreover, it surpasses the adaption to change in marketplaces and is correlated with knowledge-questioning values (Sinkula et al., 1997). The learning culture of an organization will thus be reflected in the behavioral norms that have an impact on the development and processing of various information and knowledge (Zhang and Tansuhaj, 2007).

As held by Nielsen (2005), firms with strong dynamic capability will make greater contribution to partnerships. As a result, the RE is conducive to acquiring knowledge and information and internalizing knowledge with existing PAC and RAC. As for partners, it has the spiral effect brought by continuous learning and knowledge creation. Besides, firms need to combine the latest market intelligence with their approaches and mechanisms to improve their AC (Nielsen, 2005; Escribano et al., 2009; Broersma et al., 2016). Scholars have found that RE, as a resource, has a potential value (Ketchen et al., 2007). This means appropriate learning intention and knowledge base are required to integrate firms’ AC with new knowledge acquired through RE. By virtue of strong learning attitude and culture, firms possessing sufficient AC can internalize the interfirm knowledge acquired by the enhancement of RE (Najafi-Tavani et al., 2016) and further put forward a higher level of PAC and RAC. The values of the learning culture determine the basic norms and beliefs about the reasons for and approaches of digesting, sharing, and integrating knowledge in an organization. Therefore, more LO helps members of an organization comprehend new knowledge and information, share different views, change the structure of shared meanings, and propose concrete action steps based on their understanding, thereby facilitating the development of AC. Javalgi et al. (2014) also indicate that the acquisition of new knowledge does not mean the improved quality of AC; it is easier to process external knowledge when organizational members cooperate in an open learning environment and culture (Lloréns-Montes and Molina, 2006). Based on these arguments, this study proposes the following hypotheses:

**Hypothesis 7 (H7):** Learning orientation will positively moderate relationship between RE and PAC.
**Hypothesis 8 (H8):** Learning orientation will positively moderate relationship between RE and RAC.

**FIGURE 1:**
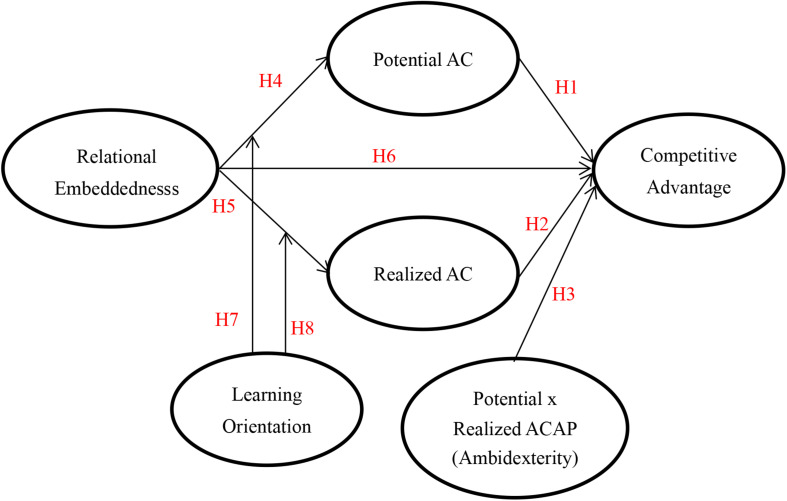
Research framework.

According to the above arguments, this study proposes Figure 1:

## Methodology

### Sampling

This study conducted a survey adopting purposive sampling. We employed Taiwanese SMEs to test the hypotheses and surveyed responses’ (top management) knowledge about their companies and their relationship with stakeholders. As these top managers should be familiar with the actual status of their firms’ internationalization, capability development, and operations, this study selected these individuals as critical information providers. We sent 1,000 questionnaires and received 214 completed surveys, giving a 21.4% response rate. There were 211 valid questionnaires after eliminating three invalid responses, giving a 21.1% effective response rate. Table 1 summarizes the respondents’ demographic characteristics.

**TABLE 1:** Demographic characteristics of respondents.

Characteristics		Frequency	Ratio
Industrial sector	Motor manufacturing	83	39.1
	Electronic parts	32	15.2
	Chemicals	23	11.1
	Semiconductors	17	8.0
	Precision machinery	17	8.0
	Information technology	29	13.5
	Other	10	5.1
Profitability	Low profit	105	50.7
	Medium profit	42	19.3
	High profit	63	30.0
Marketing proportion to total costs	<1%	20	9.4
	1–3%	61	29.1
	3–5%	49	23.2
	5–7%	46	22.0
	>10%	35	16.3
Research & Development (R&D) proportion to total costs	<1%	24	11.3
	1–3%	55	26.1
	3–5%	49	23.2
	5–7%	37	17.7
	>10%	46	21.7

This study also adopted Harman one-factor analysis method to test for common method bias (CMB). The explained variance in the first factor was 43.23%, which is smaller than the recommended threshold of 50%. Therefore, this study prevents the problems of CMB (Harman, 1976).

### Measures

Relational embeddedness is a higher-order construct. The measures of RE were drawn from Dhanaraj et al. (2004) and were measured using a 14-item scale: strong ties (3 items), trust (6 items), and shared system (3 items).

Learning orientation is a complex and higher-order construct. Following Baker and Sinkula (1999), we adopted the multidimensional methods to measure LO: four items for commitment to learning, five items for shared vision, and four items for open-mindedness.

Consistent with previous work in the management literatures (Cohen and Levinthal, 1990; Camisón and Forés, 2010), this study operationalized AC as a higher-order construct of PAC (acquisition and assimilation capacity) and RAC (transformation and application capacity). This measure consists of 19 items: acquisition (four items), assimilation (five items), transformation (five items), and application capacity (four items).

According to Gibson and Birkinshaw (2004), the concept of ambidexterity used in this work, a multiplicative term of PAC and RAC, conforms to the theoretical conceptualization of ambidexterity. Before this study measured ambidexterity as the multiplication of PAC and RAC, this study acknowledged that research results may suffer multicollinearity problems. To minimize this concern, this study mean-centered PAC and RAC deriving ambidexterity.

Competitive advantage is the direct outcomes against with competitors in foreign markets. Following Li and Zhou (2010), we adopted the multidimension to measure competitive advantage: differentiation advantage (four items), cost advantage (four items), and institutional advantage (four items). This study adopted five-point Likert scale to measure all above scales, ranging from 1 (strongly disagree) to 5 (strongly agree), as Appendix 1 showed.

## Results

### Reliability and Validity

All scales were reliable, with Cronbach’s *α* ranging from 0.770 to 0.896 (Table 2). To gauge the reliability and validity, this study adopted confirmatory factor analysis to examine construct validity (including convergent and discriminant validity). For convergent validity, following the criteria proposed by Hair et al. (2010), Table 2 shows that the average (AVE) for all dimensions was above the threshold value of 0.5. The CR values of all dimensions exceeded 0.7. Therefore, the scale had great convergent validity. The evaluation standard for discriminant validity is the square root of AVE for one dimension being greater than its correlation coefficient with any other dimension(s). As also shown in Table 2, the correlation coefficients of the dimensions were all less than the square root of AVE, suggesting that measurement model had good discriminant validity.

**TABLE 2:** Measurement of scales.

	1	2	3	4	5	6	7	8	9	10	11	12	13	14	15
(1) Size	−														
(2) Age	0.029	−													
(3) Strong ties	0.016	–0.151	0.833												
(4) Trust	0.021	–0.082	0.595	0.823											
(5) Shared	0.011	0.068	0.652	0.562	***0.707***										
(6) Acquisition	–0.062	0.023	0.279	0.171	0.214	***0.727***									
(7) Assimilation	–0.020	0.054	0.473	0.490	0.510	0.362	***0.719***								
(8) Transformation	–0.007	–0.038	0.471	0.538	0.471	0.315	0.754	***0.754***							
(9) Application	–0.034	–0.043	0.455	0.468	0.418	0.300	0.711	0.719	***0.716***						
(10) Commitment	–0.012	0.026	0.302	0.375	0.218	0.235	0.489	0.445	0.344	***0.825***					
(11) Vision	–0.003	0.038	0.361	0.380	0.312	0.332	0.465	0.446	0.377	0.724	***0.785***				
(12) Open-mind	–0.125	0.009	0.360	0.416	0.243	0.389	0.486	0.454	0.433	0.550	0.633	***0.760***			
(13) Differentiation	–0.039	–0.012	0.270	0.338	0.265	0.288	0.444	0.416	0.503	0.359	0.342	0.398	***0.731***		
(14) Cost	0.033	–0.032	0.370	0.345	0.359	0.284	0.467	0.415	0.437	0.305	0.379	0.361	0.505	***0.740***	
(15) Institution	0.169	–0.025	0.362	0.252	0.379	0.294	0.345	0.354	0.368	0.145	0.259	0.170	0.490	0.594	***0.767***
Mean	−	−	3.543	3.595	3.552	3.560	3.605	3.673	3.622	3.992	3.882	3.807	3.576	3.437	3.362
SD	−	−	0.753	0.644	0.771	0.783	0.673	0.617	0.712	0.666	0.623	0.724	0.705	0.720	0.786
α	−	−	0.896	0.851	0.887	0.832	0.843	0.814	0.807	0.879	0.877	0.835	0.770	0.827	0.843
AVE	−	−	0.694	0.678	0.500	0.529	0.517	0.569	0.513	0.681	0.616	0.577	0.534	0.548	0.589
CR	−	−	0.900	0.893	0.854	0.832	0.842	0.814	0.807	0.895	0.889	0.838	0.812	0.828	0.850

*Italicized and Bold values mean square root of AVEs.*

### Test of the Structural Model

Relational embeddedness, AC, LO, and competitive advantage are higher-order constructs, where the RE, AC, LO, and competitive advantage are measured by multiple dimensions. Relational embeddedness is conceptualized as a three-dimensional construct, LO as a three-dimensional construct, AC as a four-dimensional construct, and competitive advantage as a three-dimensional construct. Following Cadogan and Lee (2013); Özturan et al. (2014), we preferred to adopt first-order level to measure the structural model. Parcels are averages of items in a measure variable. Five constructs comprised the final structural model: competitive advantage, PAC, RAC, LO, and RE. Fit indices greater than the 0.90 benchmark (GFI = 0.97, AGFI = 0.95, TLI = 0.99, and CFI = 0.99) indicated that the data fit the said model. Additional tests included normed χ^2^ of 2.21 (less than the benchmark of 5), RMSEA = 0.045 and SRMR = 0.045 (less than benchmark of 0.08).

**FIGURE 2:**
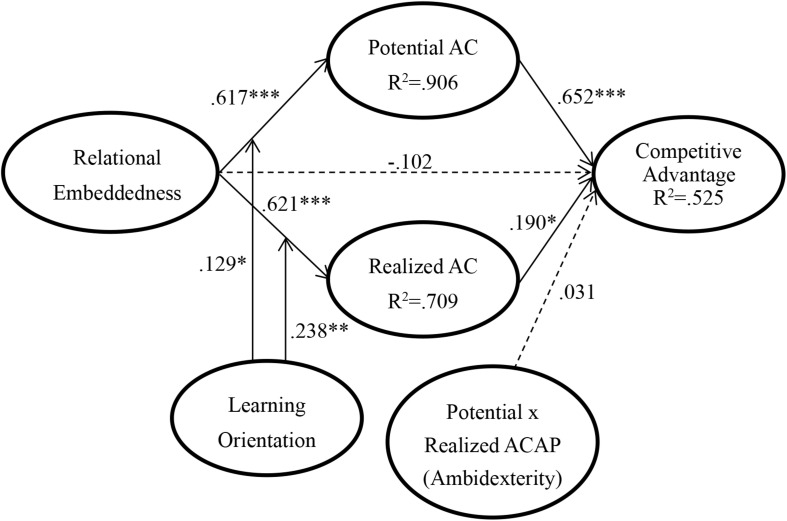
PLSSEM results.

Once we confirmed that the construct measures were reliable and valid, the next step required is to examine path of the structural model. This study performed SmartPLS version 3.2.4 to analyze data. Figure 2 shows the results of the hypothesized relationships and standardized coefficients. This study finds that RE relation has a positive effect on PAC (β = 0.617, *p* < 0.001), the RE relation would be positively associated with RAC (β = 0.621, *p* < 0.001), the PAC would be positively associated with competitive advantage (β = 0.625, *p* < 0.001), the RAC relation has a positive effect on OL (β = 0.190, *p* < 0.05), the RE relation would not be significantly associated with competitive advantage (β = −0.102, *p* > 0.05), and interaction of PAC and RAC would not be significantly associated with competitive advantage (β = 0.031, *p* > 0.05). Accordingly, H1, H2, H4, and H5 were acceptable and supported except H3. In moderating the effects of LO, the analytical results show that LO has significant moderating effects on the positive effect of RE on PAC (β = 0.129, *p* < 0.05) and RAC (β = 0.238, *p* < 0.01), thereby supporting H7 and H8.

**FIGURE 3:**
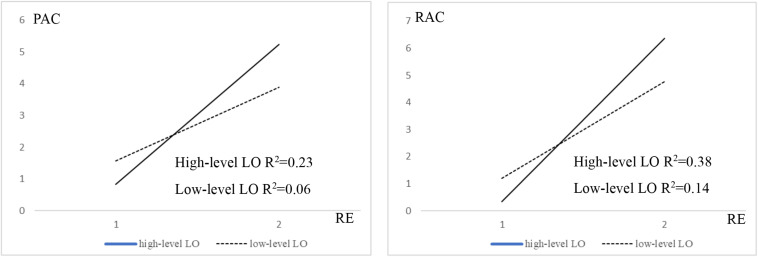
The moderation effects of high-level LO and low-level LO cultures on the links between RE and PAC and RAC.

According to the results of the moderating effect of LO, Figure 3 shows a visual depiction produced from the interaction of LO and, respectively, PAC and RAC for high-level LO and low-level LO. The *R*^2^ values indicate how much the total variation in the dependent variable (PAC and RAC) can be explained by the independent variable (RE). Accordingly, RE explains 23% of the variation of PAC for high-level LO cultures compared to 6% for low-level LO cultures. Similarly, RAC explains 37.7% of the variation of EXPI for high-level LO cultures compared to 11.4% for low-level LO cultures. Nevertheless, the *R*^2^ values are acceptable in respect of the overall model, showing a good predictive power.

### Mediation Analysis

The model proposed in this study hypothesized that AC (including PAC and RAC) would mediate the relationship between RE and competitive advantage. This study further tests for mediation following the approach proposed by Baron and Kenny (1986). In the above structural model, results indicated that the relationship between RE and competitive advantage was non-significant. Moreover, the paths of RE to AC and AC to competitive advantage were positively significant. These statistical results mean that there are mediators between RE and competitive advantage. Next, we included the mediator variable (PAC and RAC) in the PLS path model and assessed the significance of the indirect effects. The effect between RE and competitive advantage was smaller (as presented in Table 3), which indicated mediation effects. Therefore, H6 was supported.

**TABLE 3:** Results of mediation analysis.

	Mediator: PAC		Mediator: RAC	
		
Paths	RE → PAC	PAC → CA	RE → RAC	RAC → CA
Coefficients	0.371 (0.075)	0.895 (0.334)	0.617 (0.090)	0.158 (0.052)
Sobel mediation test	*Z*-value: 2.356		*Z*-value: 2.778	
Two-tailed probability	*p* < 0.001		*p* < 0.001	

## Discussion and Implications

### Discussions

This study takes social network relationship as important antecedent to cultivate internal AC of firms, which is different from the research of Liao et al. (2016); Rakthin et al. (2016), Kotabe et al. (2017), Limaj and Bernroider (2019); which considers AC as antecedent, and contrary to the research framework of Scuotto et al. (2017). Our findings show that the firms’ RE mainly and positively affects PAC and RAC (Palacios-Marqués et al., 2015a, b). As stated by Cohen and Levinthal (1990), AC enables SMEs to integrate, transfer, and assimilate knowledge and then create new insights (Zahra and George, 2002; Flatten et al., 2011; Kang and Lee, 2017; Limaj and Bernroider, 2019). In addition, this study verifies the mediating effect of AC from the social capital view, thereby supporting this argument, although previous studies indicate that organizational theory contributes to highlighting antecedents and conditions of AC (Zahra and George, 2002; Jansen et al., 2005; Lane et al., 2006; Roper et al., 2008; Limaj and Bernroider, 2019).

Further, research results show that PAC and RAC mediate the positive effects of RE on competitive advantage, while there are also direct significant positive effects of PAC and RAC on competitive advantage. Most of previous literatures, as noted earlier, indicated that there are dividing ACs into different forms (e.g., Roper et al., 2008; Limaj et al., 2016), such as PAC and RAC (Zahra and George, 2002; Kang and Lee, 2017; Flor et al., 2018; Limaj and Bernroider, 2019); external knowledge, internal knowledge, and R&D activity (Scuotto et al., 2017); and assimilation, transformation, and exploitation (Kotabe et al., 2017). Our study extends these considerations by specifically confirming the classification of PAC and RAC in the context of internationalized SMEs and further verifies the interaction of PAC and RAC. Although the interaction effect of PAC and RAC on competitive advantage is not supported, there is an ordinal relation and a reinforcing link between PAC and RAC as mentioned by scholars (Roper et al., 2008; Limaj et al., 2016; Limaj and Bernroider, 2019). Furthermore, it can be found from structural model that PAC has a higher influence on competitive advantage than RAC. This study holds that higher levels of PAC enable firms to increase their sales volume by virtue of new or substantially improved products, which is similar with the research findings of Fosfuri and Tribó (2008) and provides further support for Zahra and George’s (2002) argument that PAC serves an essential condition for realizing competitive advantage.

Our analysis indicates that LO is the moderating mechanism through which organizations build strong learning culture, corroborating the view that LO is most fundamental for consolidation and integration of various external and internal knowledge and capabilities. This research result is similar with the views of Limaj and Bernroider (2019). But the difference is that this study verifies the reinforcement effect of LO culture on developing AC, instead of using AC. Besides, this research result also extends the theoretical basis of the resource–capacity–competitive advantage (Chang and Gotcher, 2007; Murray et al., 2011) and enriches the contextual development between resource and capacity in combination with social capital and organizational learning views.

### Conclusion

Based on the literature review and hypotheses development, a research framework linking RE, AC, LO, and competitive advantage at firm level was developed and validated. In order to understand the relationship between sources of external knowledge acquisition and competitive advantage in the international context based on resource–capability–advantage process of organizational learning theory, this study, by reference to existing literatures, proposed a knowledge transformation process that AC plays a key role between them and divides into two dimensions, such as PAC and RAC. More specifically, we argued more understanding of the important role of LO in fostering PAC and RAC. Our findings indicate that RE positively affects PAC and RAC. Enterprises will more easily obtain rare and tacit knowledge from partners through close connections built through strong RE (Palacios-Marqués et al., 2015a, b), thus strengthening the basis of internal knowledge absorption and providing conducive conditions for adapting environmental changes in competition. In addition, this study verifies the mediating effects of PAC and RAC on relationship between RE and competitive advantage; this study succeeds to enrich the implications of AC using various theoretical foundations.

Moreover, this study found that PAC and RAC positively affect competitive advantage. The findings support the argument that enterprises should build on AC to drive new knowledge creation and application, as a critically important source of competitive advantage (Harris et al., 2013). Finally, our findings indicate that LO positively moderates the relationship among RE, PAC, and RAC. Specifically, results imply that internationalized SMEs that have high-level LO perform better at knowledge acquisition, classification, and application in comparison with low-level LO SMEs. Driven by LO culture, internationalized SMEs can screen and obtain knowledge and information that is conducive to the development of organizations more efficiently from social network relationship.

### Managerial Implications

According to our findings, this study has several managerial implications, particularly in answering the managerial question related to the role of international partnership and competitive advantage and the cultural conditions under which AC is useful for improving competitiveness. First, for internationalized SMEs, maintaining close relationship with partners helps to create international social communities, which generate a knowledge-based flow that has been intensively using within enterprises to introduce creative destruction (Audretsch et al., 2016). Internationalized SMEs are embedded in supply chain network (vertical network relation) and competitive network (horizontal network relation) at the same time. This study suggests internationalized SMEs to cooperate with vertical and horizontal partners via closed contacts to accumulate novel and diversified information and knowledge about demands of foreign markets and customers and then to create minimizing transaction costs and maximizing speed of international market expansion. Moreover, RE has different effects on PAC and RAC. Managers must be clear what kind of knowledge can effectively leverage and strengthen PAC or RAC, with a purpose of avoiding overinvestment when firms construct knowledge capital. To cite an example, the consumer information in real international market can be obtained through market transactions, but consumer tendency or the method breaking through the access barriers of overseas market can be obtained only through the channels constructed based on close relationship. Thus, managers should establish the criteria for knowledge classification to avoid resource loss and waste caused by repeat knowledge processing.

Second, according to this study, the RE has an impact on both PAC and RAC of internationalized SMEs. Learning orientation plays a critical strengthening mechanism between RE and AC. It can be seen that internationalized SMEs realize that they have to implement a relational sustainable strategy to reach global strategic goals. In order to create a consolidated and extensive network with stakeholders of enterprises, strategy making should be subject to limited sources. This study recommends internationalized SMEs to break through time and space constraints and build multinational and global network cooperating teams through information technology (IT)–based networking such as internet, intelligent office, and cloud computing technologies, so as to achieve development synergy of PAC and RAC that is able to promote their productivity and value creation at the lowest cost. Besides, the building of internal networking based on IT-based technology helps internal personnel communicate and connect with and learn from each other, improve the learning atmosphere in enterprises, and further enhance the absorptive efficiency of external knowledge and find out superior PAC and RAC. For example, the application of Social Networking Services (SNSs) is a motivation for employees to develop new products or services such as Zoom, Microsoft Teams, and Google Hangouts Meet.

Finally, our results show the positive impact of PAC and RAC on competitive advantage. This study focuses on critical points of managers deploying PAC and RAC, especially PAC, under the current international conditions, creating and delivering customer value, and gaining more competitive advantage than foreign competitors. Most important of all, effective AC contributes to spreading products and component design costs of a firm in many situations and launching competitive products to consumers all over the world. This study shows that PAC and RAC have achieved success in multiple situations. Therefore, managers should keep an eye on those capabilities that can be enhanced over time and that contribute to performance, such as knowledge acquisition and assimilation. Furthermore, existing AC typology may enable managers to better understand where they should pay attention and how to accelerate the capability development in order to release resources (Zahra and George, 2002; Kang and Lee, 2017; Flor et al., 2018; Limaj and Bernroider, 2019).

### Limitations and Suggestions for Future Studies

Previous studies on the direct effect of AC have been mostly discussed, but there remain gaps for exploring antecedents in formulation of AC. In addition to the RE discussed, some important variables also shape capabilities-building support sources, such as structural hole, social capital, organizational learning factor and others. In addition, although this study investigates internationalized SEMs, it does not include the influence of national cultural differences, power distance, localization, and other factors in the study variables. It is suggested that future researchers can try to add cultural differences or HRM practices to explore the formation and enhancement factors of AC. In addition to AC, the use of dynamic capability in the international business area remains a new research subject. Although AC is composed of acquisition, assimilation, transformation, and application capacity in this study, other measures are still available. There is an interesting recent study on AC that dismantles the construct of potential and realized AC (Khan et al., 2019). It would be good to include the different roles of these ACs in the Taiwanese internationalization context, and it can also be discussed in the international context of other countries in Asia.

This study is restricted to internationalized SMEs. Future research could investigate whether large Multinational enterprises (MNEs) or joint ventures utilize similar strategic behaviors. Moreover, in recent years, many studies have focused on the development and internationalization of born-global firm, and the cultivation and establishment of Dynamic internationalization Capabilities (DICs) may be different from that of general internationalized enterprises. Therefore, it is suggested that future studies focus on the DICs of born-global firm to increase its theoretical richness.

Finally, because of the cross-sectional nature of the study, we cannot test whether firms follow a sequential strategy in which they cascade their PAC into RAC or *vice versa*. To conduct such tests, additional studies could employ longitudinal data and a unit of analysis at the project level.

## Data Availability Statement

The raw data supporting the conclusions of this article will be made available by the authors, without undue reservation.

## Ethics Statement

The studies involving human participants were reviewed and approved by the Institutional Review Board, University of Taipei. The patients/participants provided their written informed consent to participate in this study.

## Author Contributions

G-SW and MP contributed to the ideas of educational research, collection of data, and empirical analysis. MP, ZC, and ZD contributed to the data analysis, design of research methods, and tables. MA and W-XZ participated in developing a research design, writing, and interpreting the analysis. All authors contributed to the literature review and conclusions.

## Conflict of Interest

The authors declare that the research was conducted in the absence of any commercial or financial relationships that could be construed as a potential conflict of interest.

## Appendix

**APPENDIX 1:** Measurement of Scale.

Construct	Variables	Items
Relational embeddedness	Strong ties	Our company received support from our foreign partners in managerial resources.
		Our company received support from our foreign partners in emotional support.
		Our company received support from our foreign partners in time.
	Trust	Because of doing business for so long, our company and partners understand each other well and quickly.
		In our contacts with the foreign partners, we have never had the feeling of being misled.
		Both sides are expected not to make demands that can seriously damage the interests of the other.
		The strongest side is expected not to pursue its interest at all costs.
		Informal agreements have the same significance as formal contracts.
		Both sides know the weaknesses of the other and do not take advantage of them.
	Shared system	The systems have been tailored to using the systems brought from the foreign partners.
		The foreign partners developed specific procedures for us to follow.
		The foreign partners have made efforts to instill its business philosophy in our managers.
Absorptive capacity	Acquisition	Capacity to capture relevant, continuous, and up-to-date information and knowledge on current and potential competitors.
		Degree of management orientation toward waiting to see what happens, instead of concern for and orientation toward their environment to monitor trends continuously and at a wide range and to discover new opportunities to be exploited proactively.
		Frequency and importance of cooperation with R&D organizations−universities, business schools, technological institutes, etc., as a member or sponsor to create knowledge and innovations.
		Effectiveness in establishing programs oriented toward the internal development of technological acquisition of competences from R&D centers, suppliers or customers.
	Assimilation	Capacity to assimilate new technologies and innovations that are useful or have proven potential.
		Ability to use employees’ level of knowledge, experience, and competencies in the assimilation and interpretation of new knowledge.
		Degree to which company employees attend and present papers at scientific conferences and congresses are integrated as lecturers at universities or business schools or receive outside staff on research attachments.
		Attendance of training courses, trade fairs, and meetings.
		Ability to develop knowledge management programs, guaranteeing the firm’s capacity for understanding and carefully analyzing knowledge and technology from other organizations.
	Transformation	Capacity of the company to use information technologies in order to improve information flow, develop the effective sharing of knowledge, and foster communication between members of the firm.
		Firm’s awareness of its competences in innovation, especially with respect to key technologies, and capability to eliminate obsolete internal knowledge, thereby stimulating the search for alternative innovations and their adaptation.
		Capacity to adapt technologies designed by other to the firm’s particular needs.
		Degree to which firm prevents all employees voluntarily transmitting useful scientific and technological information acquired to each other.
		Capability to coordinate and integrate all phases of the R&D process and its interrelations with the functional tasks of engineering, production, and marketing.
	Application	The organization’s capacity to use and exploit new knowledge in the workplace to respond quickly to environment changes.
		Degree of application of knowledge and experience acquired in the technological and business fields prioritized in the firm’s strategy that enables it to keep itself at the technological leading edge in the business.
		Capacity to put technological knowledge into product and process patents.
		Ability to respond to the requirements of demand or to competitive pressure, rather than innovating to gain competitiveness by broadening the portfolio of new products, capabilities, and technology ideas.
Competitive advantage	Differentiation advantage	Compared to competing products, our products offer superior benefits to customers.
		Our products are unique, and nobody but our company can offer them.
		We take great efforts in building a strong brand name−nobody can easily copy that.
		We successfully differentiate ourselves from others through effective advertising and promotion campaigns.
	Cost advantage	Our manufacturing costs are lower than our competitors.
		Our efficient internal operation system has decreased the cost of our products.
		Our economy of scale enables us to achieve a cost advantage.
		We have achieved a cost leadership position in the industry.
	Institutional advantage	Compared to our competitors, we have advantages in: Securing local resources such as land, electricity, and human resources; Obtaining external funding and financing; Gaining government support and approval; expediting project approval from relevant authorities.
Learning orientation	Commitment to learning	The sense around here is that employee learning is an investment, not an expense.
		The basic values of this organization include learning as key to improvement.
		Learning in my organization is seen as a key commodity necessary to guarantee organizational survival.
		Managers basically agree that our organization’s ability to learn is the key to our competitive advantage.
	Shared vision	All employees are committed to the goals of the organization.
		There is total agreement on our organizational vision across all levels, functions, and divisions.
		There is a commonality of purpose in my organization.
		Employees view themselves responsible for the direction of the organization.
		Employees view themselves as partners in charting the direction of the organization.
	Open-mindedness	Managers basically agree that it is important to accept diverse viewpoints.
		We are not afraid to reflect critically on the shared assumptions we have made about our customers.
		Our organization pays much attention to original ideas.
		The culture in our organization emphasizes continuous innovation.
